# Meta-analysis of three genome-wide association studies identifies two loci that predict survival and treatment outcome in breast cancer

**DOI:** 10.18632/oncotarget.22747

**Published:** 2017-11-28

**Authors:** Sofia Khan, Rainer Fagerholm, Latha Kadalayil, William Tapper, Kristiina Aittomäki, Jianjun Liu, Carl Blomqvist, Diana Eccles, Heli Nevanlinna

**Affiliations:** ^1^ Department of Obstetrics and Gynaecology, University of Helsinki and Helsinki University Hospital, Biomedicum, Helsinki, Finland; ^2^ Faculty of Medicine, University of Southampton, University Hospital Southampton, Southampton, UK; ^3^ Faculty of Natural and Environmental Sciences, University of Southampton, Southampton, UK; ^4^ Department of Clinical Genetics, Helsinki University Hospital and Genome Scale Biology Research Program, University of Helsinki, Helsinki, Finland; ^5^ Human Genetics, Genome Institute of Singapore, Singapore; ^6^ Yong Loo Lin School of Medicine, National University of Singapore, Singapore; ^7^ Department of Oncology, Helsinki University Hospital, Helsinki, Finland; ^8^ Department of Oncology, University of Örebro, Örebro, Sweden

**Keywords:** breast cancer, endocrine therapy, tamoxifen, GWAS, survival

## Abstract

The majority of breast cancers are driven by the female hormone oestrogen via oestrogen receptor (ER) alpha. ER-positive patients are commonly treated with adjuvant endocrine therapy, however, resistance is a common occurrence and aside from ER-status, no unequivocal predictive biomarkers are currently in clinical use. In this study, we aimed to identify constitutional genetic variants influencing breast cancer survival among ER-positive patients and specifically, among endocrine-treated patients. We conducted a meta-analysis of three genome-wide association studies comprising in total 3,136 patients with ER-positive breast cancer of which 2,751 had received adjuvant endocrine therapy. We identified a novel locus (rs992531 at 8p21.2) associated with reduced survival among the patients with ER-positive breast cancer (*P =* 3.77 × 10^−8^). Another locus (rs7701292 at 5q21.3) was associated with reduced survival among the endocrine-treated patients (*P =* 2.13 × 10^−8^). Interaction analysis indicated that the survival association of rs7701292 is treatment-specific and independent of conventional prognostic markers. *In silico* functional studies suggest plausible biological mechanisms for the observed survival associations and a functional link between the putative target genes of the rs992531 and rs7701292 (RHOBTB2 and RAB9P1, respectively). We further explored the genetic interaction between rs992531 and rs7701292 and found a significant, treatment-specific interactive effect on survival among ER-positive, endocrine-treated patients (hazard ratio = 6.97; 95% confidence interval, 1.79–27.08, *P*_interactio*n*_ *=* 0.036). This is the first study to identify a genetic interaction that specifically predicts treatment outcome. These findings may provide predictive biomarkers based on germ line genotype informing more personalized treatment selection.

## INTRODUCTION

Oestrogen receptor (ER) positive breast cancer is driven by oestrogen signalling and accounts for approximately 70% of all breast cancers [1]. ER-positive breast cancers can be successfully treated with endocrine therapies, but approximately one third do not respond to these treatments at all and the majority of tumours that initially respond to treatment will develop resistance over time [2, 3]. No biomarkers that identify such non-responders are currently in clinical use.

Both somatic mutations and germ-line variants are likely to contribute to breast cancer survival, e.g. by influencing tumour progression, host-tumour interactions, or pharmacogenomical mechanisms. Genome-wide association studies (GWASs) have investigated single nucleotide polymorphism (SNPs) associating with breast cancer survival [4], including our previous research investigating 10-year disease free survival among all breast cancer cases based on a meta-analysis of two GWAS studies [5]. Previous studies have also identified variants associating with survival in breast cancer subgroups defined by ER status [4], or by particular anticancer treatments [6]. We have previously investigated association of genetic variants with 10-year overall survival among chemotherapy-treated patients [7], and based on a two-study GWAS meta-analysis, we identified a locus 19q13.41 associating with 10-year breast cancer specific survival after endocrine treatment [8]. Our earlier studies [5, 7, 8], albeit including partly the same data sets as in the current analysis, have had different approaches on study design and sample selection. Here, our goal was to identify prognostic and predictive SNPs among patients with ER-positive tumours. To this end, we conducted a meta-analysis of SNP data from three independent GWAS studies.

## RESULTS

### SNPs at 8p21.2 are associated with survival in ER-positive breast cancer

This analysis included 3,136 patients with ER-positive breast cancer (HEBCS 513, POSH 165, SUCCESS-A 2,458) (Supplementary Figure 1 and Supplementary Table 1). The fixed-effects meta-analysis of the three study GWASs analysis was carried out using five-year follow-up for first event as defined by a combined survival endpoint of local recurrence, distant metastasis or death. Our sensitivity analysis indicated that this combined survival end-point is robust in this study (Supplementary Figures 2 and 3). In the three data sets, together 275,827 SNPs were common and passed the QC process. We conducted the analysis under additive, dominant and recessive inheritance models. The strongest meta-analysis result indicated an association with survival at a genome-wide significance level (*P* < 5 × 10^−8^) under a dominant inheritance model (Supplementary Figure 4A and 4C). The minor allele of rs992531 was associated with poor survival among ER-positive patients (hazard ratio (HR) 1.85; 95% confidence interval (CI), 1.63–2.07, *P* = 3.77 × 10^−8^) (Table 1). Two imputed SNPs, rs2314686 and rs4996307, with high LD (*r*^2^ = 1) had a slightly stronger signal (*P* = 1.70 × 10^−8^ and *P* = 3.58 × 10^−8^, respectively) (Supplementary Table 2 and Supplementary Figure 5A). The haplotype analysis of the three SNPs (rs992531, rs2314686 and rs4996307) showed that the haplotype with rare alleles for each of the SNPs had the strongest association HR = 1.71; 95% CI, 1.40–2.07, *P* = 1.91 × 10^−7^) (Supplementary Table 3). No association with survival was seen among 1,706 ER-negative patients: HR = 0.97; 95% CI, 0.74–1.28; *P* = 0.833 for the genotyped SNP rs992531 (Table 1). In a study-stratified multivariate analysis of the pooled ER-positive data set, adjusted for standard prognostic factors, rs992531 remained statistically significant (HR = 1.60; 95% CI, 1.28–2.02; *P* = 5.09 × 10^−5^) (Supplementary Table 4).

**Table 1:** Meta-analysis of univariate Cox’s regression analysis results within ER-positive patients and ER-positive endocrine treated patients (*P* < 5 × 10^−8^)

Subgroup	SNP	Location (Hg18)	MAF^a^	Survival, HEBCS GWS (MF = 5y)	Survival, POSH GWS (MF = 5y)	Survival, SUCCESS-A (MF = 4.9y)	Survival, meta-analysis
**ER-positive patients and contrast group**							
ER+	rs992531	8:23465745	0.040	1.97 (1.44–2.69)	2.07 (1.24–5.45)	1.57 (1.07–2.30)	1.85 (1.63–2.07)
				*P* = 2.32 × 10^–5^	*P* = 5.47 × 10^–3^	*P* = 0.0217	*P* = 3.77 × 10^–8^
				*N* = 513 (202)	*N* = 165 (106)	*N* = 2458 (205)	
ER-	rs992531	8:23465745	0.040	0.77 (0.46–1.31)	1.29 (0.82–2.02)	0.80 (0.47–1.36)	0.97 (0.74–1.28)
				*P* = 0.334	*P* = 0.277	*P* = 0.402	*P* = 0.833
				*N* = 230 (124)	*N* = 370 (167)	*N* = 1106 (167)	
**ER-positive endocrine treated patients and contrast groups**							
ER+, endocrine treated	rs7701292	5:105078678	0.133	2.30 (1.44–3.67)	2.13 (1.64–2.61)	1.58 (1.23–2.04)	1.79 (1.59–1.99)
				*P* = 4.58 × 10^–4^	*P* = 2.37 × 10^–3^	*P* = 4.4 × 10^–4^	*P* = 2.13 × 10^–8^
				*N* = 227 (76)	*N* = 124 (65)	*N* = 2402 (197)	
ER+, not treated with endocrine	rs7701292	5:105078678	0.133	1.02 (0.63–1.67)	1.10 (0.41–2.94)	–	–
				*P* = 0.935	*P* = 0.851		
				*N* = 247 (89)	*N* = 10 (9)		
ER-	rs7701292	5:105078678	0.133	0.93 (0.55–1.56)	1.16 (0.85–1.58)	0.88 (0.63–1.22)	0.91 (0.68–1.36)
				*P* = 0.770	*P* = 0.343	*P* = 0.437	*P* = 0.521
				*N* = 230 (124)	*N* = 370 (167)	*N* = 1106 (167)	

^a^Based on Hapmap CEU population. Study-specific MAFs are as follows for the SNP rs992531: HEBCS GWS: 0.097, POSH GWS: 0.066 and SUCCESS-A: 0.068, and for the SNP rs7701292: HEBCS GWS: 0.10, POSH GWS: 0.167 and SUCCESS-A: 0.157.

MAF = minor allele frequency; MF = median follow-up; *N* = number of samples with number of events in parenthesis. MF did not meaningfully vary between the different subgroup analyses (± 0.1y).

The survival association of rs992531 was consistent between studies in all patients and in tumour phenotype and treatment-based subgroups (Supplementary Figure 6).

### SNPs at 5p21.3 are associated with survival in patients treated with endocrine therapy

A total of 2,751 cases had received adjuvant endocrine therapy (HEBCS 227, POSH 122, SUCCESS-A 2,402) (Supplementary Figure 1 and Supplementary Table 1). The meta-analysis accepted all endocrine treated patients, combining anti-oestrogen, aromatase inhibitor and LHRH agonist treatments. The minor allele of rs7701292 was associated with poor survival after adjuvant endocrine therapy among ER-positive patients (per-allele HR = 1.79; 95% CI, 1.59–1.99, *P* = 2.13 × 10^−8^) (Table 1 and Supplementary Figures 4B, 4D and 5B). A similar effect was seen in the tamoxifen-only subgroup (HR = 1.71; 95% CI, 1.51–1.92; *P* = 2.75 × 10^−7^). No effect was seen among ER-positive patients not receiving endocrine therapy, nor among ER-negative patients (Table 1). No stronger association signals at this locus were detected in imputed genotype data (Supplementary Figure 5B). The effect of the SNP in ER-positive patients receiving endocrine therapy was consistent throughout the three studies (Table 1 and Supplementary Figure 7).

### SNP rs7701292 interacts with endocrine therapy in multivariate survival analysis

Next, we performed an interaction test between rs7701292 and endocrine treatment in a pooled data set of all ER-positive cases, adjusted for adjuvant chemotherapy and standard prognostic factors. In both the co-dominant and additive models, assuming no interaction, the SNP and endocrine treatment were both independently prognostic. In the co-dominant model, the effect size depended on allele dose: AG genotype HR = 1.44 (95% CI 1.15–1.80, *P* = 0.0015); GG genotype HR = 3.22 (95% CI 1.70–6.13, *P* = 0.0004) (Table 2). The additive model estimated the per-allele hazard at HR = 1.52, 95% CI 1.25–1.86; *P* = 3.09 × 10^−5^) (Table 2). In the models with an interaction term, a statistically significant interaction was detected between rs7701292 and endocrine treatment: the per-allele HR for rs7701292:endocrine treatment was 1.92; 95% CI, 1.11–3.31, likelihood-ratio test *P*_interaction_ = 0.0157 (Table 2).

**Table 2:** Multivariate Cox’s proportional hazards models to test for interaction between endocrine treatment and rs7701292 in the pooled data set of all ER-positive cases

Multivariate Cox’s proportional hazards models			
**Codominant model, no interaction term**			
** Covariate**	**HR**	**(95% CI)**	***P***
rs7701292 AG	1.44	(1.15–1.80)	0.0015
rs7701292 GG	3.22	(1.70–6.13)	0.0004
Endocrine treatment	0.47	(0.34–0.65)	3.81 × 10^−6^
Adjuvant chemotherapy	0.84	(0.60–1.17)	0.2941
T	1.40	(1.24–1.59)	1.8 × 10^−7^
N	2.02	(1.74–2.33)	< 10^−16^
PgR	0.71	(0.56–0.92)	0.0083
Grade	1.78	(1.50–2.12)	7.3 × 10^−11^
**Per-allele model, no interaction term**			
**Covariate**	**HR**	**(95% CI)**	***P***
rs7701292 (per allele)	1.52	(1.25–1.86)	3.09 × 10^−5^
Endocrine treatment	0.47	(0.34–0.65)	4.98 × 10^−6^
Adjuvant chemotherapy	0.83	(0.60–1.16)	0.2720
T	1.40	(1.23–1.59)	2.56 × 10^−7^
N	2.01	(1.74–2.33)	< 10^−16^
PgR	0.71	(0.55–0.91)	0.0075
Grade	1.78	(1.50–2.12)	6.58 × 10^−11^
**Per-allele model with interaction term**			
** Covariate**	**HR**	**(95% CI)**	***P***
rs7701292 (per allele)	0.89	(0.54–1.47)	0.6483
Endocrine treatment	0.39	(0.27–0.55)	1.57 × 10^−7^
Adjuvant chemotherapy	0.86	(0.62–1.19)	0.3565
T	1.41	(1.24–1.61)	1.09 × 10^−7^
N	2.01	(1.74–2.33)	< 10^−16^
PgR	0.72	(0.56–0.92)	0.0090
Grade	1.78	(1.49–2.12)	9.61 × 10^−11^
rs7701292:Endocrine	1.92	(1.11–3.31)	0.0197
** Likelihood-ratio test for interaction *P* = 0.0157**			

*N* = 2727, number of events = 369, T = tumour size; *N* = lymph node metastasis; PgR = progesterone receptor status.

We have previously identified a SNP rs8113308 at locus 19q13.14 to associate with poor survival in endocrine treated patients based on two-study meta-analysis using 10-year breast cancer specific survival [8]. In the current analysis rs8113308 did not reach genome wide significance. Additionally, we inspected whether the identified SNPs remain significant when adjusting for the SNPs found in the previous survival studies utilizing partly the same data sets as in the current analysis [5, 7, 8]. The results for the identified SNPs remain similar after adjustment (data not shown).

### Sensitivity analysis

The analyses for SNP rs7701292 and SNP rs992531 were carried out using five-year follow-up for first event as defined by a combined endpoint of local recurrence, distant metastasis or death (any cause) that was the only endpoint available for SUCCESS-A study with adequate analytic power (mean follow-up 4.0 years). However, since follow up for patients in HEBCS and POSH data sets is considerably longer (HEBCS mean follow-up 14.7 years, POSH mean follow-up 5.3 years), and also distant disease-free survival (DDFS) information is available, we carried out a sensitivity analysis for each SNP using also alternative endpoints of five year DDFS in HEBCS and POSH data sets and 10 year overall survival in all three studies (Supplementary Figures 2 and 3). The results were very similar for both SNPs for DDFS. In ER-positive patients results for rs992531 were HEBCS, HR = 1.98; 95% CI, 1.65–2.29 and POSH, HR = 2.21; 95% CI, 1.69 - 2.72, and in the subgroup of ER-positive patients who received endocrine treatment results for rs7701292 were, HEBCS, HR = 2.32; 95% CI, 1.83–2.81 and POSH, HR = 2.07; 95% CI, 1.57–2.56. (Supplementary Figures 2 and 3). The same trend was also seen for the less powered 10 year overall survival analysis in the subgroup of ER-positive patients for rs992531 and among ER-positive endocrine treated patients for rs7701292 (Supplementary Figures 2 and 3).

### Association with clinical predictors

The SNP rs992531 was associated with M-status (metastasis at diagnosis) in the ER positive subgroup: carriers of the rare allele had more metastases at diagnosis than carriers of the common homozygote genotype (*P* = 0.004) (Supplementary Table 5). The rare homozygote of rs7701292 also associated with a higher rate of metastasis at diagnosis, but only among ER-negative cases (*P* = 0.003) (Supplementary Table 6).

Neither of the SNPs were associated with ER-status (rs99253: χ^2^ = 0.31, *P* = 0.58 and rs7701292: χ^2^ = 0.44, *P* = 0.80).

### eQTL analysis

In order to test whether SNP rs992531 in chromosome 8 or rs7701292 in chromosome 5 or their tag SNPs in the linkage disequilibrium region (*r*^2^ > 0.1) associate with mRNA expression, we performed a quantitative expression trait (eQTL) analysis in 1142 breast cancer tumours in the METABRIC project [9, 10]. We conducted the analysis both in *cis*, defined as eQTL within ±1 Mb from any SNP in the LD region, and *trans*, defined as any eQTLs outside these regions.

SNP rs992531 resides in a gene-dense region with 55 genes. The analysis utilizing a linear model identified two *cis*-eQTL genes, *PDLIM2* and *RHOBTB2,* which remained statistically significant after multiple testing correction (Supplementary Table 7). No *trans*-eQTLs were observed at this locus.

In the *cis*-region of rs7701292, the SNP rs2061968 (a tag SNP for rs7701292; *r*^2^ = 1) associated with the expression of *RAB9P1* (*P* = 3.55 × 10^−2^) (Supplementary Table 8). Low *RAB9P1* mRNA expression level was associated with the rs2061968 rare homozygote genotype. Rs7701292 and its tag SNPs were also associated with seven statistically significant *trans*-eQTL genes (Supplementary Table 9).

### *In silico* functional studies

In the rs992531 locus, the SNP rs4996307 (*r*^2^ = 1) is located in the binding motif of the transcription factor AP-1, a predicted regulator of *RHOBTB2* [11]. The genomic region spanning the binding motif is active in several cell lines including human mammary epithelial cell line and MCF-7 breast cancer cell line indicated by the histone modification data [12]. Inspection of haplotypes at the rs992531 locus revealed that the survival-associated haplotypes contain the rare alleles of rs992531 and rs4996307 (Supplementary Table 10). In the rs7701292 locus, we found a binding motif for hypoxia-inducible factor 1β (HIF-1β) that is affected by rs1712662 (tag SNPs for rs7701292, *r*^2^ = 0.79 D’ = 1). As indicated by the histone modification data, the genomic region spanning the binding motif for HIF-1β is active in several cell lines including MCF-7 breast cancer cell line [12]. HIF-1β targets *RAB9P1* in MCF-7 breast cancer cells [13].

### Gene expression and survival

According to Kaplan-Meier Plotter [14], one of the two *cis*-eQTL genes associating with rs992531, *RHOBTB2,* was associated with survival consistently with the association seen in the eQTL analysis. Low expression of the *RHOBTB2* gene was associated with poor prognosis among ER positive patients (HR = 1.17; 95% CI 1.05–1.31; *P* = 0.0038) whereas among ER-negative patients the effect was opposite (HR = 0.87; 95% CI 0.77–0.98; *P* = 0.02) (Supplementary Figure 8). This is consistent with the results of the *cis*-eQTL analysis: the heterozygote and rare homozygote genotypes of rs1550281 (a tag SNP for rs992531, *r*^2^ = 0.414, D’ = 1) were associated with decreased *RHOBTB2* expression, which in turn was associated with poor survival.

The only identified *cis*-eQTL association in the 5q21.3 locus was *RAB9P1*. In line with the eQTL result, high *RAB9P1* expression was associated with better survival (HR = 0.80, 95% CI 0.71–0.91, *P* = 8.1 × 10^−4^). The effect was similar in ER-positive cases, HR = 0.75; 95% CI 0.61–0.92, *P* = 5.7 × 10^−3^ and in ER-negative cases, HR = 0.73; 95% CI 0.56–0.97, *P* = 0.028, indicating that the association between *RAB9P1* expression and survival is not specific to the ER-positive subgroup. Since the *cis*-eQTL *RAB9P1* gene is a pseudogene, and a pseudogene may affect the function of its cognate protein coding gene [15], we also investigated its parent gene, *RAB9A* [16]. In the Kaplan-Meier plotter analysis, high *RAB9A* expression was associated with poor survival among patients with ER-positive cancers who received endocrine treatment (HR = 2.05 (1.48–2.83), *P* = 8.6 × 10^−6^) whereas an opposite effect was seen among ER-negative cases (HR = 0.70 (0.53–0.94), *P* = 0.015) (Supplementary Figure 9).

### Rs992531 and rs7701292 have an interactive effect on survival

Finally, in order to evaluate a possible biological connection between the two loci (see Discussion), we performed an exploratory interaction test between the minor allele of rs992531 and the rare homozygote of rs7701292, and found a statistically significant interaction specifically within the ER-positive endocrine treated group (interaction term HR 6.97, 95% CI 1.79–27.08, *P*_interaction_ = 0.036) (Supplementary Table 11).

## DISCUSSION

We have identified one new locus (rs992531 at 8p21.2) associated with poor survival in the ER-positive subgroup and a second locus (rs7701292 at 5q21.3) that is prognostic specifically among endocrine-treated patients. In a multivariate analysis adjusted for adjuvant chemotherapy and conventional prognostic factors, we found an interaction between rs7701292 and endocrine treatment indicating a predictive, treatment-specific effect of the SNP on breast cancer survival.

**Figure 1:**
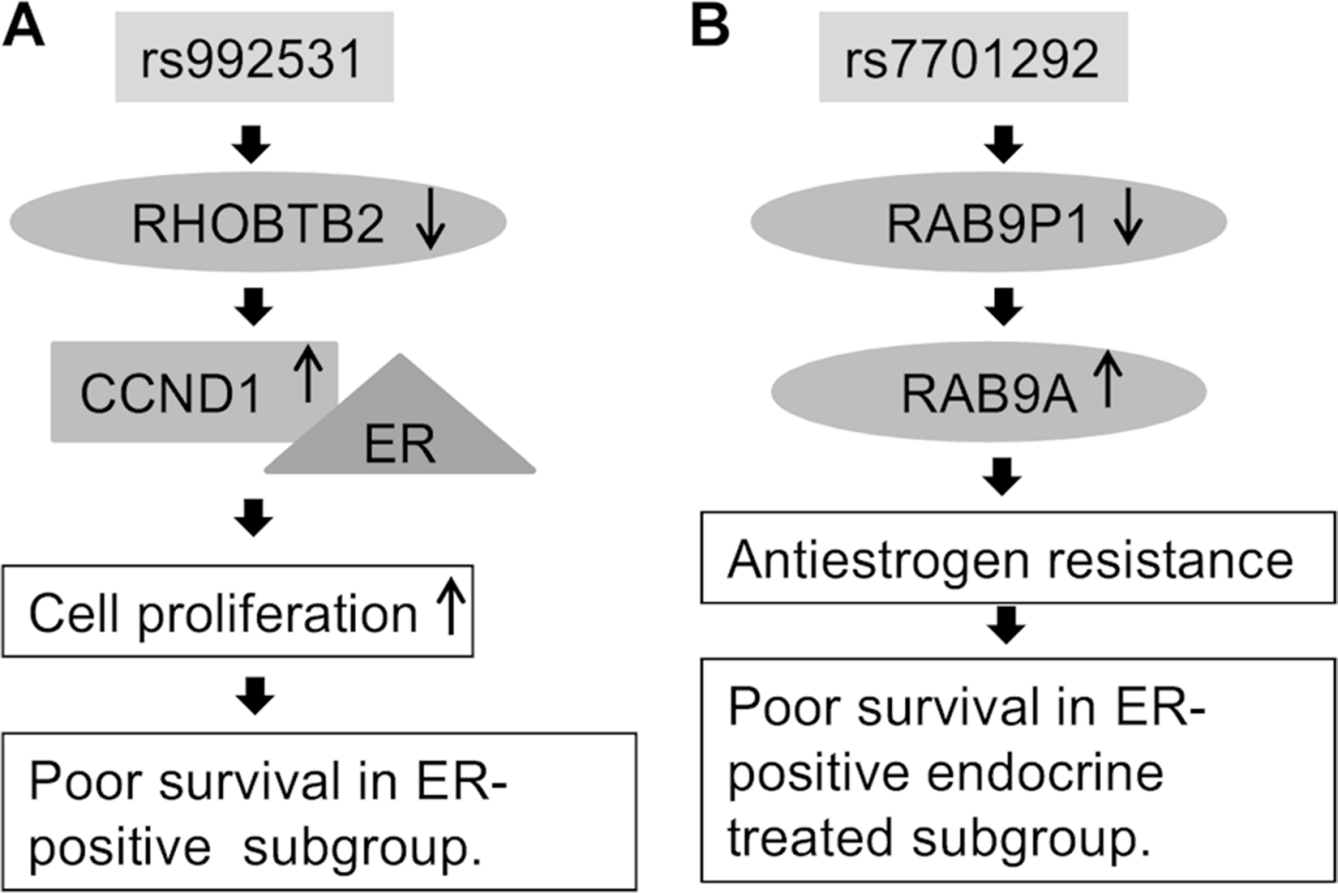
Putative biological mechanism of (**A**) SNP rs992531 and (**B**) rs7701292.

In the rs992531 locus, the statistically most significant imputed SNP (rs4996307) resides in the binding motif of the transcription factor AP-1, predicted to be the main transcriptional regulator of *RHOBTB2*. Our results indicate that the rs992531 minor allele associates with decreased expression of *RHOBTB2* which, in turn, associates with poor survival in ER-positive breast cancer patients. *RHOBTB2* has been implicated as a tumour suppressor gene associated with breast cancer survival [17, 18]. Its growth-inhibitory effect may be mediated via down-regulation of cyclin D1 [19] which binds the oestrogen receptor and has been shown to associate with poor prognosis in ER-positive breast cancer [20, 21]. Cyclin D1 and its associated cyclin-dependent kinases (CDKs) are potential therapeutic targets: improved survival has been achieved with a combination therapy of CDK4/6 inhibitor palbociclib and fulvestrant, an ER antagonist, compared to fulvestrant alone [22]. The molecular mechanism behind SNP rs992531 and poor breast cancer outcome in ER-positive cases may therefore involve altered *RHOBTB2* expression and its interplay with cyclin D1 and the oestrogen receptor (Figure 1A).

In the rs7701292 locus, the minor allele was found to associate with decreased expression of *RAB9P1*. Rs1712662, a tag SNP for rs7701292 (*r*^2^ = 0.79 D’ = 1), disrupts a binding motif for HIF-1β, a regulator of *RAB9P1* in the ER-positive MCF-7 breast cancer cell line [13]. *RAB9P1* is a pseudogene that shares high sequence homology with its parent/cognate protein coding gene *RAB9A*, and may influence its function [15]. Rab GTPases have central roles in tumour progression and the development of drug resistance, and are dysregulated in many cancers, including breast cancer [23]. Rab GTPases, including RAB9A, are also involved in the regulation of autophagy, a biological process known to facilitate the progression of ER-positive breast cancer cells to anti-oestrogen resistance [24–26]. In KM-plotter data, high expression of *RAB9A* was associated with poor survival in the ER-positive subgroup, and the ER-positive endocrine treated subgroup, whereas an opposite effect was seen in the ER-negative subgroup. The biological rationale for the rs7701292 association might thus be that under-expression of *RAB9P1*, possibly due to impaired HIF-1β mediated regulation, may upregulate *RAB9A* expression or activity, leading to anti-oestrogen resistance and consequent poor survival (Figure 1B).

There appears to be a possible functional link between the rs992531 and rs7701292 loci and their putative target genes *RHOBTB2* and *RAB9A*. RHOBTB2 belongs to the RhoBTB family and RAB9A binds another member of the same family, RHOBTB3. RHOBTB2 and RHOBTB3 are capable of dimerizing through the BTB domain region [27]. Both proteins participate in vesicle transport and interact with CUL3, a ubiquitin E3 ligase that co-activates ER. Downregulation of *RHOBTB2* and *RHOBTB3* is common in breast cancer [27]. Based on this connection, we further explored the SNPs in the two identified loci, rs992531 and rs7701292, and found an interaction on survival between minor allele of rs992531 and the rare homozygote genotype of rs7701292 specifically within the ER-positive endocrine treated patient group. Certain caution is advisable in the interpretation of this result, however, as ER-positive endocrine-treated patients carrying the interacting genotypes represent only 0.2% of cases in the study.

In conclusion, we have identified two SNPs associated with survival at genome wide significance level, one among patients with ER positive breast cancer (rs992531) and one associated with outcome specifically after endocrine treatment (rs7701292), independently of conventional prognostic factors and consistently across the studies. If validated in other large studies, these findings help identify patients unlikely to benefit from current approaches to adjuvant endocrine treatment. This is also the first study to detect a possible genetic interaction on patient survival that may specifically predict treatment outcome with potentially major clinical consequences for the patients.

## MATERIALS AND METHODS

### Genotype data

GWAS data was obtained from three studies with well characterized patient series: HEBCS, POSH and SUCCESS-A.

The HEBCS GWS consisted of 805 cases, of which 563 originated from a prospective patient series of unselected, incident breast cancer patients and 242 from an ongoing collection of familial cases. The POSH GWS consisted of 536 participants from the prospective POSH study in which participants were diagnosed with invasive breast cancer aged 40 years or younger. SUCCESS-A is a sub-study of the Simultaneous Study of Gemcitabine-Docetaxel Combination adjuvant treatment, as well as Extended Bisphosphonate and Surveillance-Trial. The sample set used here consisted of 3,596 samples. All participants of the three studies provided written informed consent as part of the original study. Ethical approval was obtained from institutional review boards of the contributing centers. See Supplementary Table 1 for details on patient and tumour characteristics and Supplementary Materials and Methods for a detailed description of case recruitment, genotyping, quality control, and genotype imputation.

### Statistical analysis and *in silico* methods

Cox’s proportional hazards models were used to analyse survival until first event as defined by a combined endpoint of local recurrence, distant metastasis or death (any cause), and adjusted for BRCA1/2 and age. The additive, dominant, and recessive genetic models were analysed. For the endocrine treatment subgroup, we combined anti-oestrogen, aromatase inhibitor and LHRH agonist treatments. Follow-up time was calculated from the date of diagnosis to the date of last follow-up or local recurrence, distant metastasis or death (any cause) and right-censored at five years. Patients with distant metastases near the time of diagnosis (M1) were excluded from the treatment-specific analyses.

Likelihood ratio tests were used to test for interaction between genotype and endocrine treatment in a pooled data set of all three studies, stratified by study. Clinically relevant covariates (tumour size [T], lymph node metastasis [N], progesterone receptor [PgR] status, age at diagnosis, tumour grade, and adjuvant chemotherapy treatment) were included in these Cox models. The interaction analyses were performed using both co-dominant (genotype-specific) and additive (per-allele) genetic models. Association between genotypes and clinical features was evaluated using chi-square tests.

To detect eQTL genes likely to be affected by variants discovered in this study, we utilized breast tumour data from METABRIC [9, 10]. HaploReg4 was used for regulatory element prediction [28]. To assess gene expression-based survival we utilized the online database Kaplan–Meier –Plotter [14]. See Supplementary Materials and Methods for more details.

## SUPPLEMENTARY MATERIALS FIGURES AND TABLES



## References

[1] Harvey JM, Clark GM, Osborne CK, Allred DC (1999). Estrogen receptor status by immunohistochemistry is superior to the ligand-binding assay for predicting response to adjuvant endocrine therapy in breast cancer. J Clin Oncol.

[2] Riggins RB, Bouton AH, Liu MC, Clarke R (2005). Antiestrogens, aromatase inhibitors, and apoptosis in breast cancer. Vitam Horm.

[3] Osborne CK, Schiff R (2011). Mechanisms of endocrine resistance in breast cancer. Annu Rev Med.

[4] Guo Q, Schmidt MK, Kraft P, Canisius S, Chen C, Khan S, Tyrer J, Bolla MK, Wang Q, Dennis J, Michailidou K, Lush M, Kar S (2015). Identification of novel genetic markers of breast cancer survival. J Natl Cancer Inst.

[5] Rafiq S, Khan S, Tapper W, Collins A, Upstill-Goddard R, Gerty S, Blomqvist C, Aittomäki K, Couch FJ, Liu J, Nevanlinna H, Eccles D (2014). A genome wide meta-analysis study for identification of common variation associated with breast cancer prognosis. PLoS One.

[6] Kiyotani K, Mushiroda T, Tsunoda T, Morizono T, Hosono N, Kubo M, Tanigawara Y, Imamura CK, Flockhart DA, Aki F, Hirata K, Takatsuka Y, Okazaki M (2012). A genome-wide association study identifies locus at 10q22 associated with clinical outcomes of adjuvant tamoxifen therapy for breast cancer patients in Japanese. Hum Mol Genet.

[7] Fagerholm R, Schmidt MK, Khan S, Rafiq S, Tapper W, Aittomäki K, Greco D, Heikkinen T, Muranen TA, Fasching PA, Janni W, Weinshilboum R, Loehberg CR (2015). The SNP rs6500843 in 16p13.3 is associated with survival specifically among chemotherapy-treated breast cancer patients. Oncotarget.

[8] Khan S, Fagerholm R, Rafiq S, Tapper W, Aittomäki K, Liu J, Blomqvist C, Eccles D, Nevanlinna H (2015). Polymorphism at 19q13.41 Predicts Breast Cancer Survival Specifically after Endocrine Therapy. Clin Cancer Res.

[9] Curtis C, Shah SP, Chin SF, Turashvili G, Rueda OM, Dunning MJ, Speed D, Lynch AG, Samarajiwa S, Yuan Y, Graf S, Ha G, Haffari G (2012). The genomic and transcriptomic architecture of 2,000 breast tumours reveals novel subgroups. Nature.

[10] Dvinge H, Git A, Graf S, Salmon-Divon M, Curtis C, Sottoriva A, Zhao Y, Hirst M, Armisen J, Miska EA, Chin SF, Provenzano E, Turashvili G (2013). The shaping and functional consequences of the microRNA landscape in breast cancer. Nature.

[11] Park CY, Krishnan A, Zhu Q, Wong AK, Lee YS, Troyanskaya OG (2015). Tissue-aware data integration approach for the inference of pathway interactions in metazoan organisms. Bioinformatics.

[12] Consortium EP (2012). An integrated encyclopedia of DNA elements in the human genome. Nature.

[13] Lachmann A, Xu H, Krishnan J, Berger SI, Mazloom AR, Ma’ayan A (2010). ChEA: transcription factor regulation inferred from integrating genome-wide ChIP-X experiments. Bioinformatics.

[14] Gyorffy B, Lanczky A, Eklund AC, Denkert C, Budczies J, Li Q, Szallasi Z (2010). An online survival analysis tool to rapidly assess the effect of 22,277 genes on breast cancer prognosis using microarray data of 1,809 patients. Breast Cancer Res Treat.

[15] Poliseno L, Salmena L, Zhang J, Carver B, Haveman WJ, Pandolfi PP (2010). A coding-independent function of gene and pseudogene mRNAs regulates tumour biology. Nature.

[16] Sisu C, Pei B, Leng J, Frankish A, Zhang Y, Balasubramanian S, Harte R, Wang D, Rutenberg-Schoenberg M, Clark W, Diekhans M, Rozowsky J, Hubbard T (2014). Comparative analysis of pseudogenes across three phyla. Proc Natl Acad Sci U S A.

[17] Mao H, Zhang L, Yang Y, Sun J, Deng B, Feng J, Shao Q, Feng A, Song B, Qu X (2011). RhoBTB2 (DBC2) functions as tumor suppressor via inhibiting proliferation, preventing colony formation and inducing apoptosis in breast cancer cells. Gene.

[18] Mao H, Qu X, Yang Y, Zuo W, Bi Y, Zhou C, Yin H, Deng B, Sun J, Zhang L (2010). A novel tumor suppressor gene RhoBTB2 (DBC2): frequent loss of expression in sporadic breast cancer. Mol Carcinog.

[19] Yoshihara T, Collado D, Hamaguchi M (2007). Cyclin D1 down-regulation is essential for DBC2’s tumor suppressor function. Biochem Biophys Res Commun.

[20] Zwijsen RM, Wientjens E, Klompmaker R, van der Sman J, Bernards R, Michalides RJ (1997). CDK-independent activation of estrogen receptor by cyclin D1. Cell.

[21] Aaltonen K, Amini RM, Landberg G, Eerola H, Aittomäki K, Heikkilä P, Nevanlinna H, Blomqvist C (2009). Cyclin D1 expression is associated with poor prognostic features in estrogen receptor positive breast cancer. Breast Cancer Res Treat.

[22] Turner NC, Ro J, Andre F, Loi S, Verma S, Iwata H, Harbeck N, Loibl S, Huang Bartlett C, Zhang K, Giorgetti C, Randolph S, Koehler M (2015). Palbociclib in Hormone-Receptor-Positive Advanced Breast Cancer. N Engl J Med.

[23] Recchi C, Seabra MC (2012). Novel functions for Rab GTPases in multiple aspects of tumour progression. Biochem Soc Trans.

[24] Nozawa T, Aikawa C, Goda A, Maruyama F, Hamada S, Nakagawa I (2012). The small GTPases Rab9A and Rab23 function at distinct steps in autophagy during Group A Streptococcus infection. Cell Microbiol.

[25] Ao X, Zou L, Wu Y (2014). Regulation of autophagy by the Rab GTPase network. Cell Death Differ.

[26] Schoenlein PV, Periyasamy-Thandavan S, Samaddar JS, Jackson WH, Barrett JT (2009). Autophagy facilitates the progression of ERalpha-positive breast cancer cells to antiestrogen resistance. Autophagy.

[27] Berthold J, Schenkova K, Ramos S, Miura Y, Furukawa M, Aspenstrom P, Rivero F (2008). Characterization of RhoBTB-dependent Cul3 ubiquitin ligase complexes--evidence for an autoregulatory mechanism. Exp Cell Res.

[28] Ward LD, Kellis M (2012). HaploReg: a resource for exploring chromatin states, conservation, and regulatory motif alterations within sets of genetically linked variants. Nucleic Acids Res.

