# Supplementary material for: Meta-analysis of three genome-wide association studies identifies two loci that predict survival and treatment outcome in breast cancer
Source: Oncotarget. 2017 Nov 28;9(3):4249–57. doi: 10.18632/oncotarget.22747 (PMC5790536; doi:10.18632/oncotarget.22747)
Supplement: Supplementary file 1 [file oncotarget-09-4249-s001.pdf]

Meta-analysis of three genome-wide association studies identifies two loci that predict survival and treatment outcome in breast cancer

SUPPLEMENTARY MATERIALS

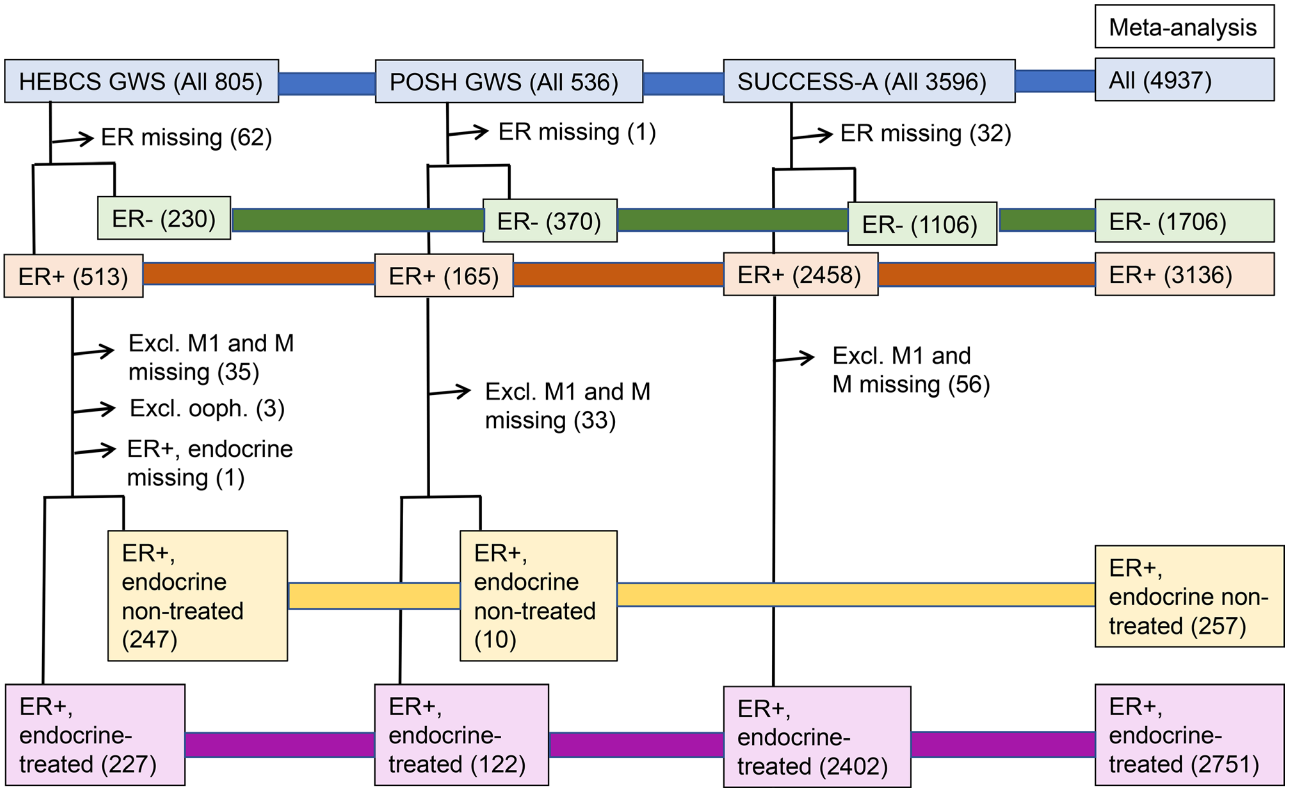

Supplementary Figure 1: REMARK diagram describing the work flow and sample selection in this study.

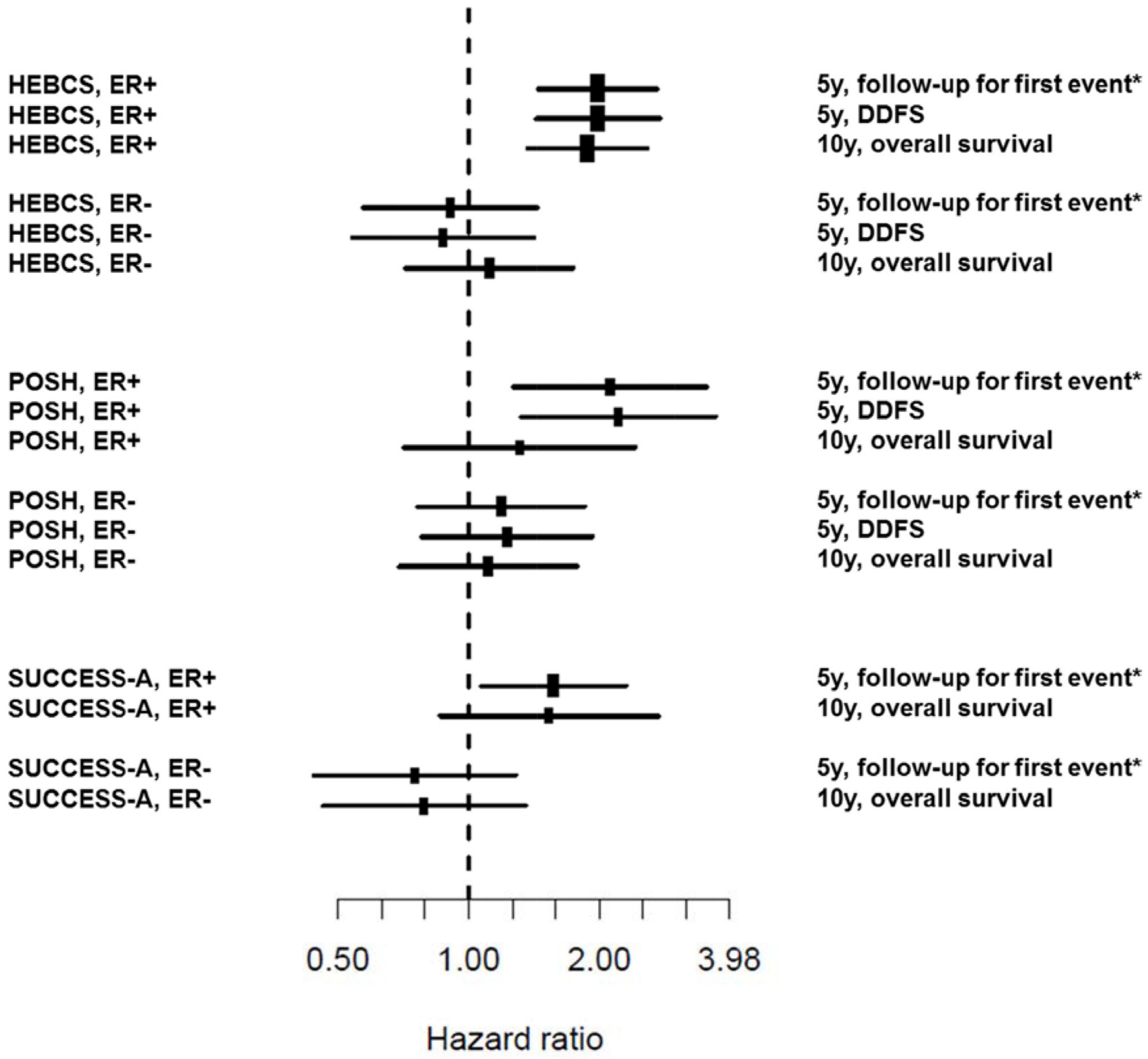

Supplementary Figure 2: Sensitivity analysis for the SNP rs992531. Forest plots of hazard ratios and their confidence intervals separately for HEBCS, POSH and SUCCESS-A. The main analysis has been conducted using five year follow up for first event defined as a combined endpoint of local recurrence, distant metastasis or death (any cause). In addition the hazard ratios and their confidence intervals are shown for five year follow-up using distant disease-free survival (DDFS) (HEBCS and POSH) and 10 year overall survival (all three studies) in ER positive subgroup and ER negative subgroup. *a combined endpoint of local recurrence, distant metastasis or death (any cause).

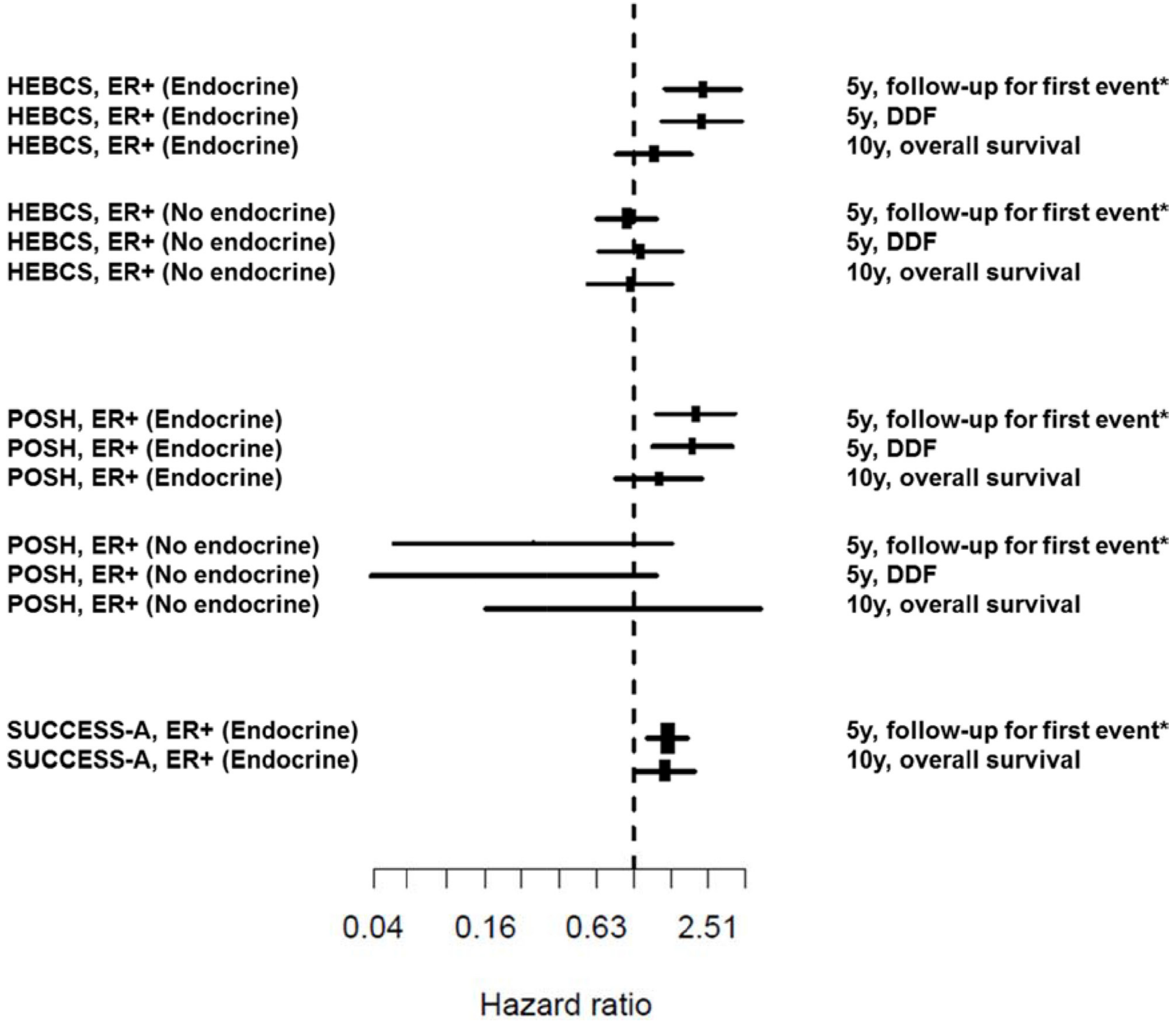

Supplementary Figure 3: Sensitivity analysis for the SNP rs7701292. Forest plots of hazard ratios and their confidence intervals separately for HEBCS, POSH and SUCCESS-A. The main analysis has been conducted using five year follow up for first event defined as a combined endpoint of local recurrence, distant metastasis or death (any cause). In addition the hazard ratios and their confidence intervals are shown for five year follow-up using distant disease-free survival (DDFS) (HEBCS and POSH) and 10 year overall survival (all three studies) in ER positive endocrine treated subgroup and ER positive subgroup (HEBCS and POSH). *a combined endpoint of local recurrence, distant metastasis or death (any cause).

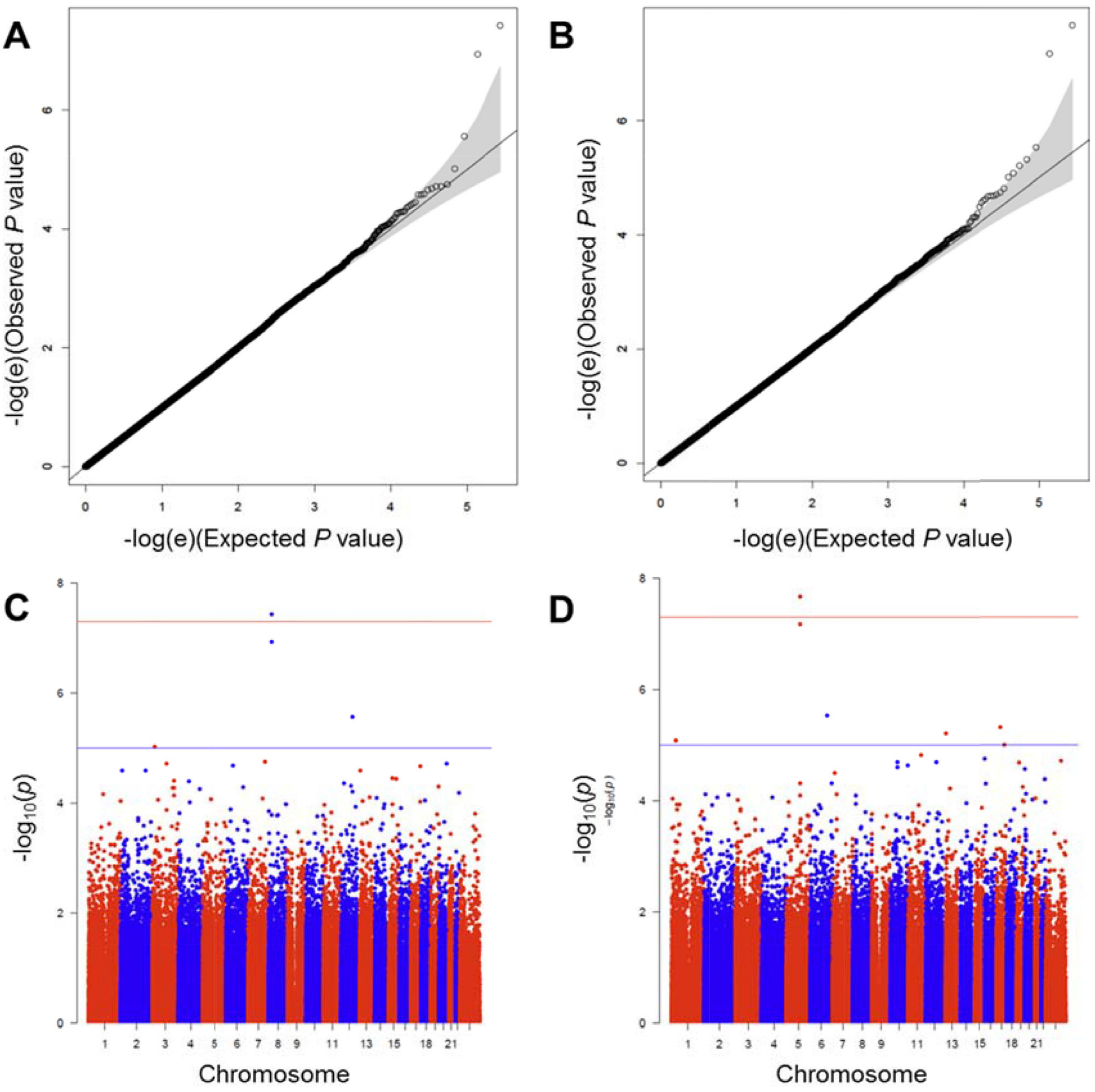

Supplementary Figure 4: Quantile-quantile and Manhattan plots. Quantile-quantile plots of the observed vs. expected chi-squared statistics and Manhattan plots for the strength of genetic association versus chromosomal position for the meta-analysis results of genotyped SNPs. (**A**) Quantile-quantile plot for association in ER-positive subgroup ($\lambda = 1.00$). (**B**) Quantile-quantile plot for associations in ER-positive, endocrine treated subgroup ($\lambda = 1.01$). (**C**) Manhattan plot for associations in ER-positive subgroup. (**D**) Manhattan plot for associations in ER-positive, endocrine treated subgroup. In quantile-quantile plots each circle represents the chi-squared statistic for a single variant. The black diagonal line represents the predicted association statistics under the global null hypothesis of no association. In Manhattan plots each dot represents a single variant. Red horizontal line corresponds to the $P = 5 \times 10^{-8}$, blue horizontal line corresponds to $P = 10^{-5}$.

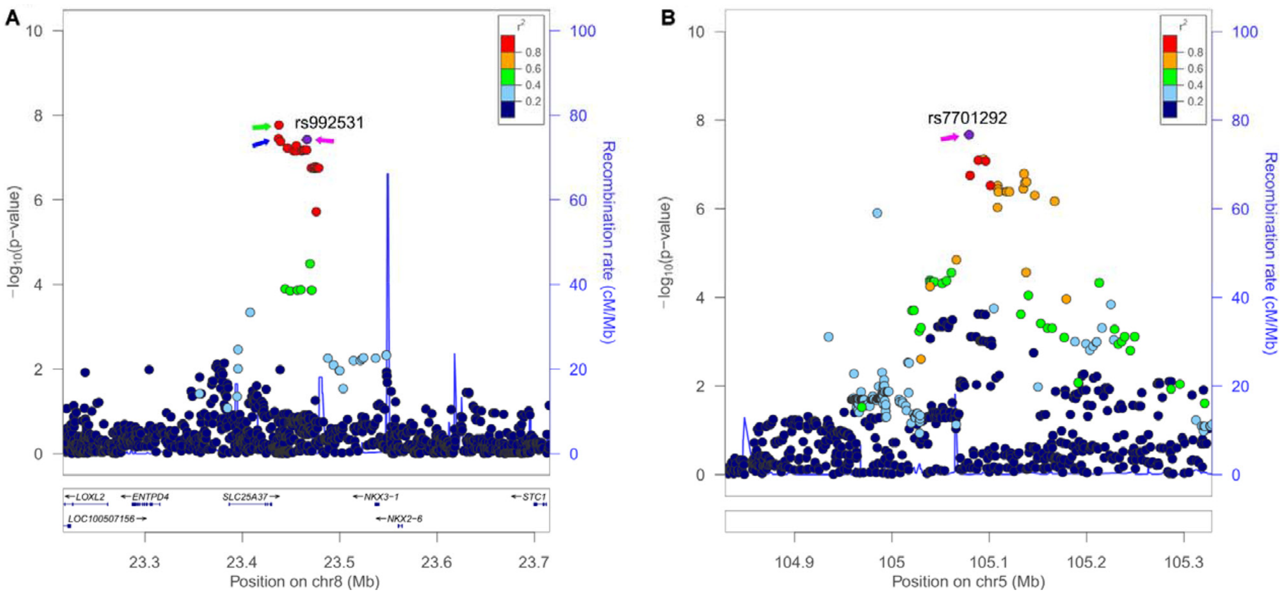

Supplementary Figure 5: Regional plots of meta-analysis *P* values. Regional plot using P values derived from univariate Cox's regression model from HEBCS, POSH and SUCCESS-A meta-analysis and including both imputed and the genotyped SNPs 250 kb either side of (**A**) rs992531 (pointed with pink arrow). The imputed SNPs rs2314686 and rs4996307 are pointed with green and blue arrows, respectively) and (**B**) rs7701292 (pointed by pink arrow).

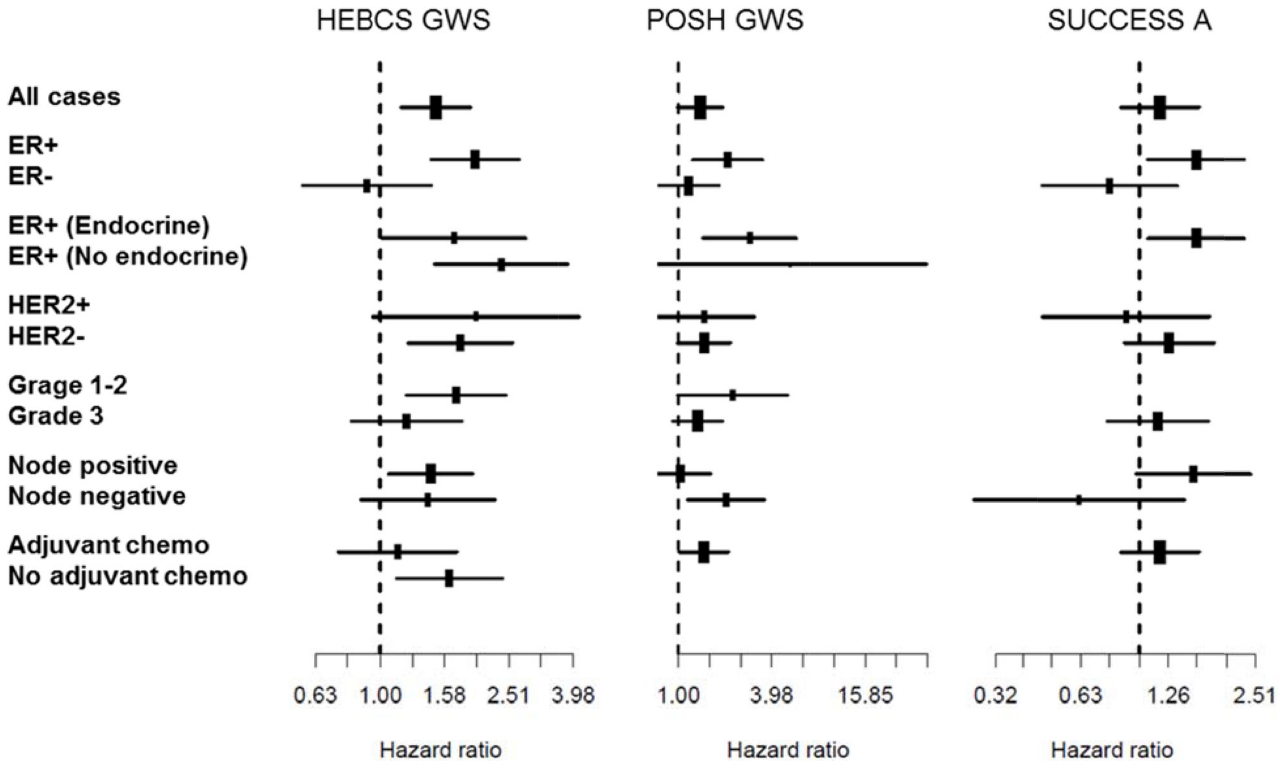

Supplementary Figure 6: Forest plots of hazard ratios and their confidence intervals for the SNP rs992531 separately in all samples and within phenotype- and treatment-based subgroups in each of the three studies.

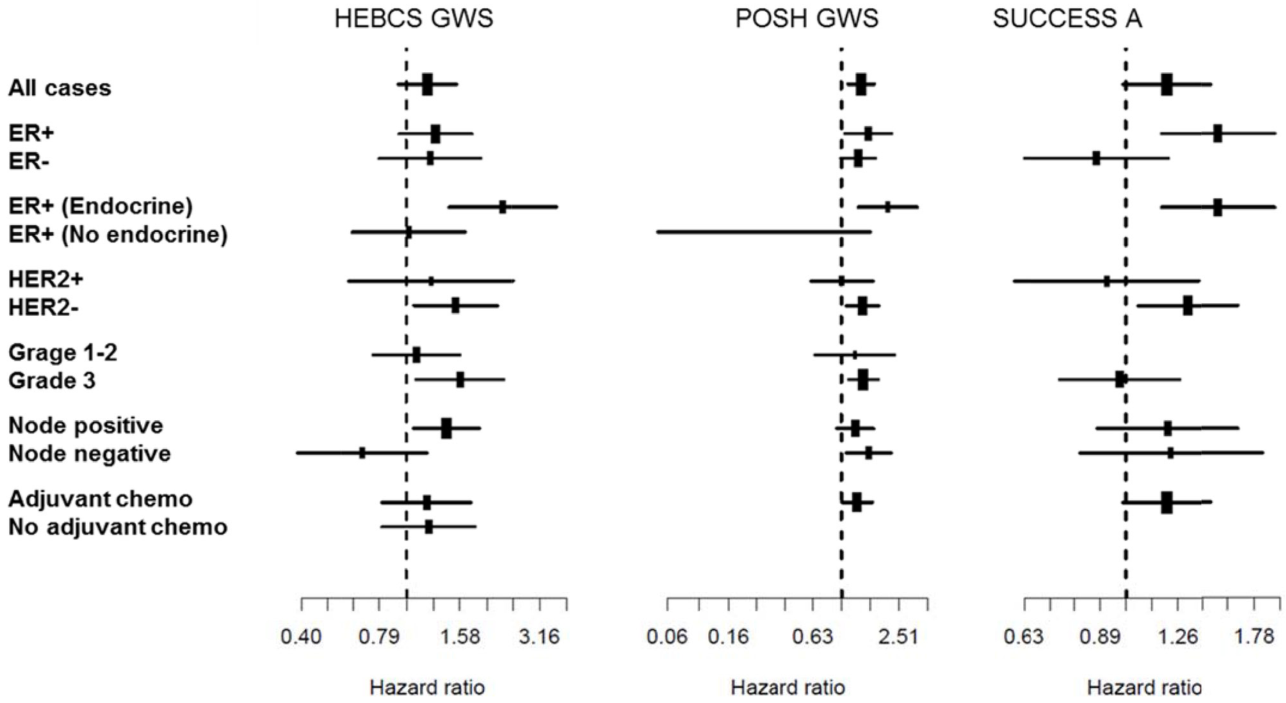

Supplementary Figure 7: Forest plots of hazard ratios and their confidence intervals for the SNP rs7701292 separately in all samples and within phenotype- and treatment-based subgroups in each of the three studies.

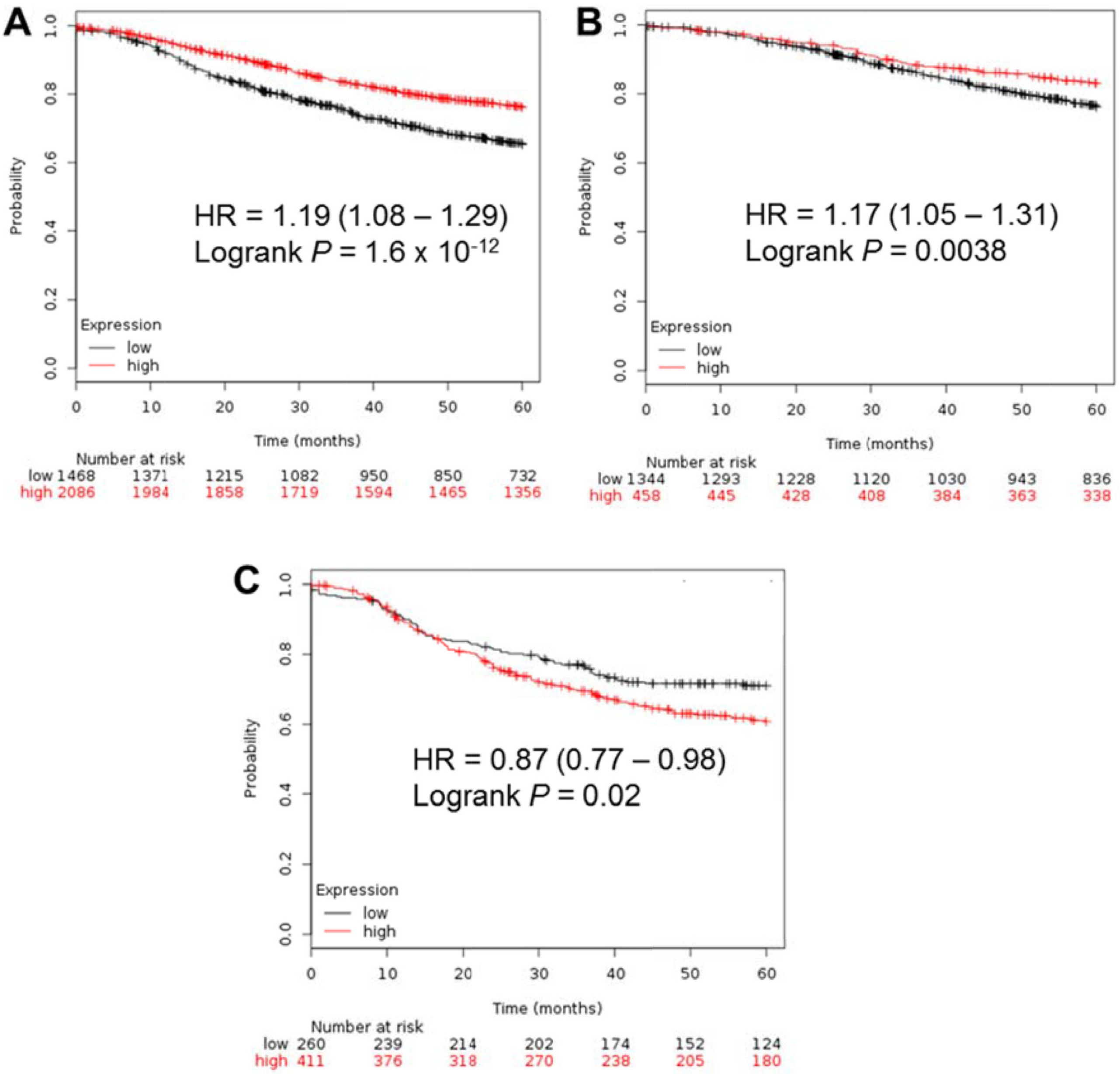

Supplementary Figure 8: Gene expression survival curves with relapse free survival as endpoint and five-year follow-up time for RHOBTB2 (probe set: 209441_at) obtained from Kaplan-Meier Plotter database in (**A**) all cases, (**B**) ER-positive cases and (**C**) ER-negative cases.

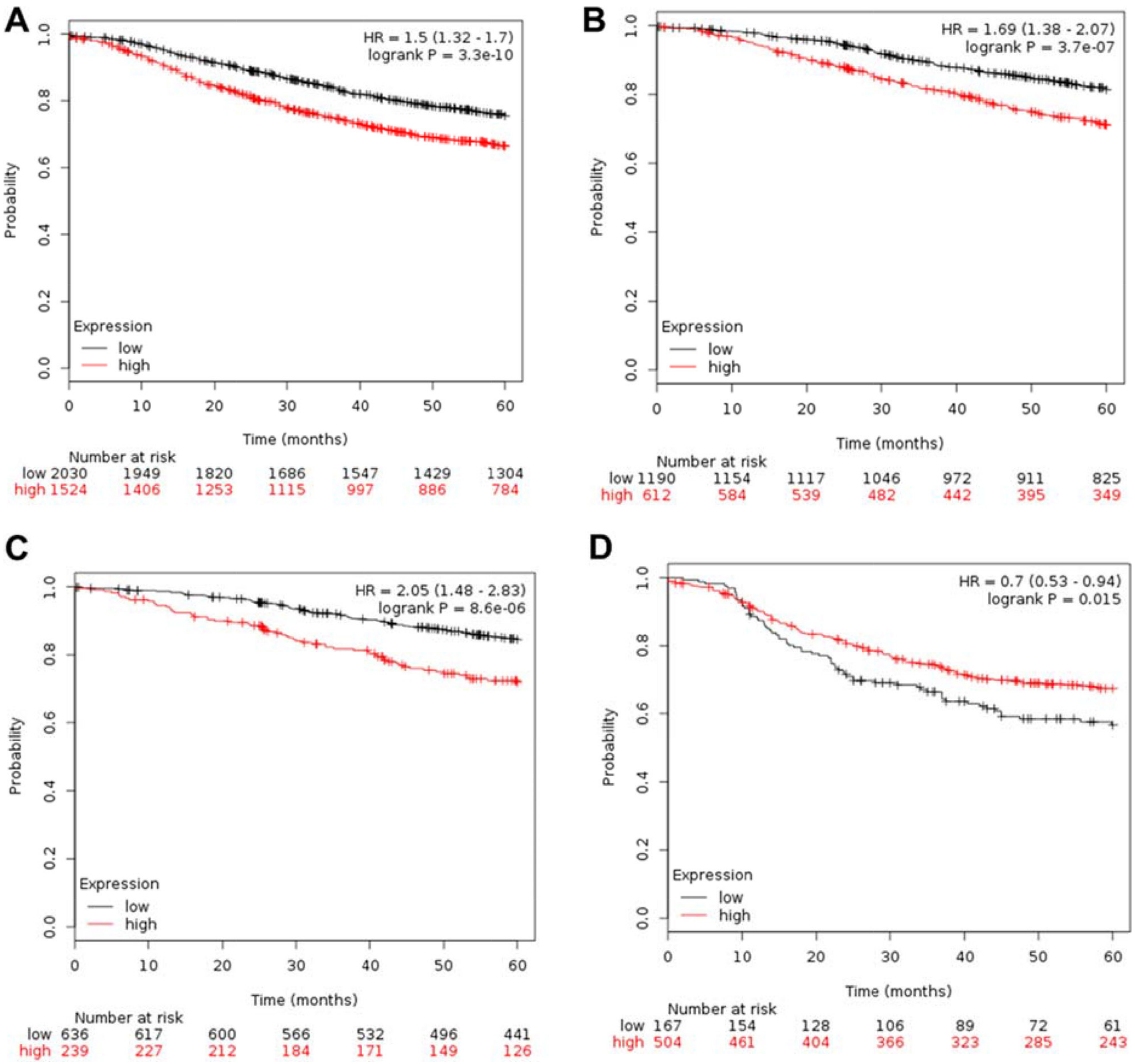

Supplementary Figure 9: Gene expression survival curves with relapse free survival as endpoint and five-year follow-up time for RAB9A (probe set: 221808_at) obtained from the Kaplan-Meier plotter database in (**A**) all cases, (**B**) ER-positive cases and (**C**) ER-positive endocrine treated cases and (**D**) ER-negative cases.

Supplementary Table 1: Age and tumor characteristics of study participants from HEBCS GWS, POSH GWS, and SUCCESS-A

Characteristics	HEBCS GWS	POSH GWS	SUCCESS-A
**No. of cases**	805	536	3596
**Vital status**			
**Alive**	466 (58%)	300 (56%)	3389 (94%)
**Deceased: all-cause**	339 (42%)	236 (44%)	207 (6%)
**Deceased: BC-specific**	312 (39%)	235 (44%)	NA
**Follow-up mean ± SD (only censored)**	14.7 ± 4.9	5.3 ± 1.7	4.0 ± 1.7
**Age, mean [range],y**	54.1 [22– 87]	35.8 [18– 41]	53.6 [19– 85]
**ER**			
**Negative**	230 (29%)	370 (69%)	1106 (31%)
**Positive**	513 (64%)	165 (31%)	2458 (68%)
**Missing, No.**	62 (8%)	1 (0.2%)	32 (1%)
**Grade**			
**1**	144 (18%)	13 (2%)	165 (5%)
**2**	312 (39%)	84 (16%)	1698 (47%)
**3**	280 (35%)	422 (79%)	1698 (47%)
**Missing, No.**	69 (9%)	17 (3%)	35 1%)
**T**			
**1**	390 (48%)	232 (43%)	1464 (41%)
**2**	304 (38%)	236 (44%)	1856 (52%)
**3**	50 (6%)	49 (9%)	192 (5%)
**4**	47 (6%)	12 (2%)	50 (1%)
**Missing, No.**	14 (2%)	7 (1%)	34 (1%)
**N**			
**Negative**	338 (42%)	248 (46%)	1248 (35%)
**Positive**	446 (55%)	262 (49%)	2311 (64%)
**Missing, No.**	21 (3%)	26 (5%)	37 (1%)
**M**			
**Negative**	740 (92%)	481 (90%)	3487 (97%)
**Positive**	57 (7%)	50 (9%)	4 (0.1%)
**Missing, No.**	8 (1%)	5 (1%)	105 (2.9%)
**Adjuvant chemotherapy treatment**[a]	364 (45%)	518 (96.6%)	3596 (100%)
**A&T**	14 (2%)	129 (24%)	-
**Antracyclines**	191 (24%)	376 (70%)	3596 (100%)
**Taxanes**	2 (0.2%)	8 (1.5%)	-
**CMF**	153 (19%)	4 (1%)	-
**Adjuvant Endocrine treatment**[a,b]	227 (28.2%)	122 (22.8%)	2402 (68%)
**Anti-estrogen (Tamoxifen)**	214 (27%)	118 (22%)	2402 (68%)
**Aromatase inhibitor**	13 (1.6%)	3 (0.6%)	223 (6%)
**LHRH agonist**	-	33 (6.2%)	29 (1%)
**No endocrine treatment (tamoxifen/AI/LHRH agonist)**	247 (31%)	10 (1.9%)	-

[a]In the adjuvant chemotherapy/endocrine treatment subgroups, the total numbers may not add up, since a patient may have received several types of adjuvant chemotherapy/endocrine treatments. bIn the adjuvant endocrine treatment subgroups the numbers include ER-positive patients excluding M1 and missing M-status samples as well as samples with only partial oophorectomy information. Abbreviations in the table: NA = not available, T = tumor size according to TNM classification, *N* = metastasis to lymph node, M = distant metastasis.

Supplementary Table 2: Meta-analysis of univariate Cox's regression analysis results of imputed SNPs within ER-positive patients ($P < 5 \times 10^{-8}$)

Subgroup	SNP	Location	MAF	HEBCS GWS (HR (95% CI)) [MF = 5y]	POSH GWS (HR (95% CI)) (MF = 5y)	SUCCESS-A (HR (95% CI)) (MF = 4.9y)	Meta-analysis (HR (95% CI))
ER+	rs2314686 [imp]	8:23436752	0.035	2.07 (1.75–2.39) $P = 8.14 \times 10^{-6}$ $N = 506$ (202)	2.06 (1.55–2.56) $P = 4.94 \times 10^{-3}$ $N = 165$ (106)	1.64 (1.28–1.99) $P = 6.23 \times 10^{-3}$ $N = 2265$(178)	1.89 (1.66–2.11) $P = 1.70 \times 10^{-8}$
ER+	rs4996307 [imp]	8:23436382	0.037	1.92 (1.60–2.23) $P = 4.54 \times 10^{-5}$ $N = 506$ (202)	2.06 (1.55–2.56) $P = 4.94 \times$ 10–3 $N = 165$ (106)	1.70 (1.35–2.05) $P = 2.94 \times 10^{-3}$ $N = 2265$(178)	1.84 (1.63–2.06) $P = 3.58 \times 10^{-8}$
ER-	rs2314686 [imp]	8:23436752	0.035	0.95 (0.48–1.41) $P = 0.818$ $N = 224$ (122)	1.21 (0.78–1.64) $P = 0.389$ $N = 370$ (167)	0.81 (0.28–1.35) $P = 0.447$ $N = 1017$ (148)	1.00 (0.73–1.28) $P = 0.984$
ER-	rs4996307 [imp]	8:23436382	0.037	0.92 (0.46–1.37) $P = 0.702$ $N = 224$ (122)	1.21 (0.78–1.64) $P = 0.389$ $N = 370$ (167)	0.80 (0.27–1.33) $P = 0.413$ $N = 1017$ (148)	0.99 (0.72–1.26) $P = 0.918$

Abbreviations in the table: MAF = minor allele frequency, imp = imputed, N = number of samples with number of events in parenthesis, MF = median follow-up of the five-year follow-up for first event as defined by a combined endpoint of local recurrence, distant metastasis or death (any cause) among the individuals with censored data. MF did not meaningfully vary between the different subgroup analyses (± 0.1y).
The table presents per study as well as the meta-analysis results within ER-positive patients and ER-negative patients. The imputed SNPs rs2314686 and rs4996307 are tag SNPs for rs992531 with $r^2 = 1$ and D′= 1.

Supplementary Table 3: Univariate Cox's regression analysis results for the haplotypes for the top SNP hits (rs992531, imputed SNP rs4996307 and imputed SNP rs2314686) in the rs992531 locus

Haplotypes	HR (95%CI)	*P* value	hap.freq	rs992531	rs4996307	rs2314686
HAP1 [reference]	.	.	0.9258	1	1	1
HAP2	0.13 (0.02–0.83)	0.877	0.0001	1	1	2
HAP3	1.37 (0.54–3.47)	0.651	0.0013	1	2	2
HAP4	0.1 (0.05–0.22)	0.544	0.0012	2	1	1
HAP5	1.05 (0.4–2.75)	0.92	0.0023	2	2	1
HAP6	1.71 (1.4–2.07)	1.91E-07	0.0693	2	2	2

In the genotype columns, 1 = major allele, 2 = minor allele.

Supplementary Table 4: Multivariate Cox's proportional hazards model in the pooled data set of ER-positive cases of HEBCS and POSH GWS, and SUCCESS-A

Covariate	HR (95% CI)	*P* value
per-allele rs992531	1.60 (1.28–2.02)	5.09×10^{-5}
T	1.37 (1.22–1.54)	1.18×10^{-7}
N	1.87 (1.63–2.15)	$< 2.00 \times 10^{-16}$
Grade	1.66 (1.42 – 1.94)	1.50×10^{-10}

The model was stratified by study and adjusted for tumour size, lymph node metastasis, tumour histological grade, and age at diagnosis.

Supplementary Table 5: Association analysis between SNP rs992531 and clinical predictors

	ER+					ER-				
**rs992531**	**G/G**		**G/A+A/A**			**G/G**		**G/A+A/A**		
	n	**(%)**	***n***	**(%)**	***P***	***n***	**(%)**	***n***	**(%)**	***P***
**Grade**										
**1**	240	*10%*	34	*8%*	0.518	18	*1%*	4	*1%*	0.39
**2**	1401	*56%*	231	*56%*		263	*19%*	46	*21%*	
**3**	840	*34%*	150	*36%*		1081	*79%*	168	*77%*	
**Tumor size**										
**1**	1051	*42%*	179	*43%*	0.133	612	*44%*	97	*44%*	0.865
**2**	1255	*50%*	196	*47%*		659	*48%*	102	*47%*	
**3**	154	*6%*	25	*6%*		82	*6%*	14	*7%*	
**4**	52	*2%*	17	*4%*		25	*2%*	5	*2%*	
**Nodal satus**										
**Negative**	723	*35%*	111	*32%*	0.435	751	*60%*	112	*56%*	0.243
**Positive**	1328	*65%*	239	*68%*		499	*40%*	89	*44%*	
**Metastasis at diagnosis**										
**Negative**	2429	*98%*	391	*96%*	0.004	1332	*98%*	208	*97%*	0.346
**Positive**	51	*2%*	18	*4%*		30	*2%*	7	*3%*	
**Pr status**										
**Negative**	368	*15%*	59	*14%*	0.779	1235	*91%*	195	*91%*	0.852
**Positive**	2098	*85%*	351	*86%*		127	*9%*	21	*9%*	
**Her2**										
**Negative**	1724	*74%*	278	*74%*	0.711	843	*66%*	137	*69%*	0.316
**Positive**	592	*26%*	96	*26%*		429	*34%*	59	*31%*	

Supplementary Table 6: Association analysis between SNP rs7701292 and clinical predictors

	ER+							ER-						
**rs7701292**	**A/A**		**A/G**		**G/G**			**A/A**		**A/G**		**G/G**		
	N	**(%)**	***N***	**(%)**	***N***	**(%)**	***P***	***N***	**(%)**	***N***	**(%)**	***N***	**(%)**	***P***
**Grade**														
**1**	202	*8%*	68	*9%*	4	*7%*	0.604	17	*1%*	4	*1%*	1	*3%*	0.263
**2**	1176	*56%*	428	*57%*	28	*49%*		214	*19%*	91	*23%*	4	*11%*	
**3**	711	*34%*	257	*34%*	25	*44%*		915	*80%*	308	*76%*	30	*86%*	
**Tumor size**														
**1**	907	*43%*	297	*39%*	26	*46%*	0.442	512	*44%*	183	*45%*	16	*46%*	0.949
**2**	1028	*49%*	397	*52%*	29	*50%*		559	*48%*	188	*47%*	16	*46%*	
**3**	130	*6%*	48	*6%*	1	*2%*		69	*6%*	24	*6%*	3	*8%*	
**4**	48	*2%*	20	*3%*	1	*2%*		21	*2%*	9	*2%*	0	*0%*	
**Nodal satus**														
**Negative**	616	*35%*	205	*33%*	14	*28%*	0.366	615	*58%*	229	*62%*	21	*64%*	0.378
**Positive**	1123	*65%*	408	*67%*	37	*72%*		439	*42%*	139	*38%*	12	*36%*	
**Metastasis at diagnosis**														
**Negative**	2033	*97%*	734	*98%*	56	*98%*	0.675	1130	*98%*	382	*96%*	32	*91%*	0.003
**Positive**	53	*3%*	15	*2%*	1	*2%*		19	*2%*	15	*4%*	3	*9%*	
**Pr status**														
**Negative**	293	*14%*	121	*16%*	13	*23%*	0.09	1044	*91%*	359	*89%*	31	*89%*	0.315
**Positive**	1782	*86%*	626	*84%*	44	*77%*		102	*9%*	42	*11%*	4	*11%*	
**Her2**														
**Negative**	1435	*74%*	529	*75%*	40	*70%*	0.756	710	*67%*	245	*64%*	27	*77%*	0.181
**Positive**	491	*26%*	180	*25%*	17	*30%*		343	*33%*	139	*36%*	8	*23%*	

Supplementary Table 7: Genes in the rs992531 *cis*-region with *cis*-eQTL results at *P* value < 0.05

Gene symbol	Gene name	mRNA-data available	Tag SNP	r2	D'	beta	t-stat	*P* value	Benjamini-Hochberg corrected *P* value	Gene type
PDLIM2	PDZ and LIM domain 2 (mystique)	yes	rs992531	1	1	0.043	3.742	1.90E-04	4.00E-03	protein coding
RHOBTB2	Rho-related BTB domain containing 2	yes	rs1550281	0.41	1	0.081	3.444	5.92E-04	6.21E-03	protein coding
PPP3CC	protein phosphatase 3, catalytic subunit, gamma isozyme	yes	rs4871881	0.32	1	0.092	2.505	0.012	0.054	protein coding
SLC25A37	solute carrier family 25 (mitochondrial iron transporter), member 37	yes	rs2872716	0.32	1	0.157	2.49	0.013	0.054	protein coding
CHMP7	charged multivesicular body protein 7	yes	rs11992418	1	1	0.069	2.199	0.028	0.084	protein coding

Supplementary Table 8: Genes in the rs7701292 *cis*-region

Gene symbol	Gene name	mRNA-data available	Tag SNP	*r*2	D’	*F*-test	*P* value	Gene type
SNORA31		no	.	.	.	.	.	.
RNU6-334P		no	.	.	.	.	.	.
RNA5SP189		no	.	.	.	.	.	.
RP11-6N13.1		no	.	.	.	.	.	.
RP11-6N13.4		no	.	.	.	.	.	.
RAB9BP1	RAB9B, member RAS oncogene family pseudogene 1	yes	rs2061968	1	1	3.347	0.0355	pseudogene
CTD-2374C24.1		no	.	.	.	.	.	.
CTC-278L1.1		no	.	.	.	.	.	.
CTD-2285G11.1		no	.	.	.	.	.	.
CTC-254B4.1		no	.	.	.	.	.	.

Supplementary Table 9: *Trans*-eQTL genes with significant Benjamini-Hochberg corrected *P* values for rs7701292 locus

Gene symbol	Gene name	Tag SNP	r^2	D’	*F*-test	*P* value	Benjamini-Hochberg corrected *P* value	Gene type
CALCA	calcitonin-related polypeptide alpha	rs13165324	0.62	0.82	29.635	2.57E-13	2.76E-07	protein coding
INSM2	insulinoma-associated 2	rs13165324	0.62	0.82	15.847	1.58E-07	1.57E-02	protein coding
USP16	ubiquitin specific peptidase 16	rs4957841	0.57	0.82	15.359	2.54E-07	2.19E-02	protein coding
LHX8	LIM homeobox 8	rs286706	0.79	1.00	15.318	2.65E-07	2.19E-02	protein coding
FBXO34	F-box protein 34	rs286737	0.51	1.00	15.122	3.21E-07	2.46E-02	protein coding
CEP89	centrosomal protein 89kDa	rs10050771	0.33	1.00	14.567	5.52E-07	3.71E-02	protein coding
CSRP1	cysteine and glycine rich protein 1	rs286737	0.51	1.00	14.242	7.59E-07	4.53E-02	protein coding

Supplementary Table 10: Univariate Cox's regression analysis results for the haplotypes in the rs992531 locus

Haplotypes	HR (95%CI)	*P* value	hap.freq	rs4996307	rs11992418	rs28619160	rs992531	rs12549446	rs925490	rs907584	rs1550281	rs4621817
HAP1 [reference]	.	.	0.863	1	1	1	1	1	1	1	1	1
HAP2	1.90 (1.57–2.23)	1.40E-04	0.019	2	2	2	2	2	1	1	1	1
HAP3	1.53 (1.29–1.77)	4.50E-04	0.053	2	2	2	2	2	2	2	2	2
HAP4	0.77 (0.33–1.21)	0.25	0.030	1	1	2	1	2	1	1	1	1
HAP5	0.80 (0.32–1.27)	0.35	0.025	1	1	1	1	1	2	2	2	2
Haplotypes with frequency < 0.01 combined	0.69 (−0.18–1.56)	0.4	< 0.01	.	.	.	.	.	.	.	.	.

In the genotype columns, 1 = major allele, 2 = minor allele.

Supplementary Table 11: Multivariate Cox's proportional hazards models to test for interaction between rs992531 and rs7701292 among ER-positive endocrine treated cases (in the pooled dataset of HEBCS and POSH GWS and SUCCESS-A); among ER-positive endocrine non-treated cases (in the pooled dataset of HEBCS and POSH GWS); and among ER-negative cases (in the pooled dataset of HEBCS and POSH GWS and SUCCESS-A)

**ER-positive endocrine treated cases**	**HR (95% CI)**	***P*-value**
**Model assuming no interaction**		
rs7701292 A/G	1.72 (1.36–2.18)	6.81E-06
rs7701292 G/G	2.97 (1.56–5.65)	9.43E-04
rs992531 G/A + A/A	1.69 (1.27–2.25)	3.20E-04
Model with interaction term		
rs7701292 A/G	1.67 (1.28–2.16)	1.28E-04
rs7701292 G/G	1.92 (0.84–4.38)	1.20E-01
rs992531 G/A + A/A	1.47 (1.01–2.15)	4.43E-02
rs7701292 A/G:rs992531 G/A + A/A	1.20 (0.66–2.19)	5.46E-01
rs7701292 G/G:rs992531 G/A + A/A	6.97 (1.79–27.08)	5.05E-03
Likelihood ratio test *P* value	**3.64E-02**	
**ER-positive endocrine non-treated cases**	**HR (95% CI)**	***P* value**
**Model assuming no interaction**		
rs7701292 A/G	0.93 (0.57 –1.52)	7.79E-01
rs7701292 G/G	NA	NA
rs992531 G/A + A/A	2.30 (1.45–3.63)	3.68E-04
**Model with interaction term**		
rs7701292 A/G	0.81 (0.45 –1.46)	4.74E-01
rs7701292 G/G	NA	NA
rs992531 G/A + A/A	2.01 (1.16–3.48)	1.22E-02
rs7701292 A/G:rs992531 G/A + A/A	1.62 (0.58–4.47)	3.55E-01
rs7701292 G/G:rs992531 G/A + A/A	NA	NA
Likelihood ratio test *P* value	**3.58E-01**	
**ER-negative cases**	**HR (95% CI)**	***P* value**
**Model assuming no interaction**		
rs7701292 A/G	1.10 (0.88 –1.37)	4.02E-01
rs7701292 G/G	1.36 (0.72 –2.56)	3.42E-01
rs992531 G/A + A/A	0.97 (0.73–1.27)	8.00E-01
**Model with interaction term**		
rs7701292 A/G	1.05 (0.83 –1.34)	6.92E-01
rs7701292 G/G	1.29 (0.66 –2.65)	4.49E-01
rs992531 G/A + A/A	0.88 (0.63–1.23)	4.45E-01
rs7701292 A/G:rs992531 G/A + A/A	1.35 (0.74–2.47)	3.24E-01
rs7701292 G/G:rs992531 G/A + A/A	1.74 (0.21–14.11)	6.05E-01
Likelihood ratio test *P* value	**5.72E-01**	

SUPPLEMENTARY MATERIALS AND METHODS

Study population

For HEBCS GWS, 805 cases were included. Of these, 423 cases originated from a prospective patient series of unselected, incident breast cancer patients treated in the Helsinki University Central Hospital Department of Oncology in years 1997–1998 and 2000 [1, 2] as well as 140 cases collected 2001-2004 and 242 additional familial cases [3]. All familial cases and the majority of non-familial cases had been tested negative for BRCA1 and BRCA2 [3]. Among all the patients genotyped, 6% were found to carry BRCA1 or BRCA2 mutation, for 30% of samples the BRCA status had not been tested. The GWS series was specifically enriched for cases with reduced survival (i.e. distant metastasis or death at the time of the initiation of the study in 2008).

The POSH GWS consisted of 536 participants from the POSH study [4] in which participants were diagnosed with invasive breast cancer aged 40 years or younger. Recruitments to the POSH cohort were made between January 2000 and January 2008 from oncology clinics across the UK. The vast majority (98%) of patients recruited to the study presented symptomatically. The detailed description of the POSH study participants is presented elsewhere [4]. Sample selection for POSH GWS was enriched for patients with either very short (<2 years) survival or relatively long (>4 years) survival and included patients with triple negative breast cancer (ER, progesterone receptor (PR) and HER2 receptor negative) who have poor prognosis and early relapse after diagnosis. Additionally, the sample selection comprised of patients. Sample selection for POSH GWS is described in detail in [5]. Among all the patients genotyped in POSH GWS, 7% were found to carry BRCA1 or BRCA2 mutation while for 80% of the samples the BRCA status had not been tested.

As a third data set, we used SUCCESS-A, a sub-study of the Simultaneous Study of Gemcitabine-Docetaxel Combination adjuvant treatment, as well as Extended Bisphosphonate and Surveillance-Trial, data that is available through the database of Genotypes and Phenotypes (dbGaP) [6]. The sample set used here consisted of 3,596 samples of the sample series of 3,754 patients that were recruited from 2005 to 2007 from 250 study sites across Germany.

Genome-wide genotyping and harmonized quality control of HEBCS and POSH GWS

Genotyping of the HEBCS and POSH samples were conducted using illumina platform (Illumina 550 and Illumina 660-Quad SNP array, respectively) as previously described [5, 7]. The intensity files of both data sets were processed with Illumina's Genome Studio software using a GenCall threshold of 0.15. SNPs with a MAF<0.01, a genotyping call rate <95% and Hardy-Weinberg equilibrium (HWE) P value < 1 × 10-4 were excluded from the analysis. The detailed description of quality control has been previously described [5].

Imputation of HEBCS, POSH and SUCCESS-A genotypes

The imputation of genome wide SNP information in HEBCS and POSH GWS was performed based on 1000 Genomes Project phase 1 and release version 3 European reference haplotypes with program MaCH (http://www.sph.umich.edu/csg/abecasis/MACH/index.html). Quality control measures applied to imputed data included excluding SNPs with MAF<0.02, imputed genotype call rate ≤ 90% and HWE P value < 1 × 10−6 and individuals call rate ≤ 90%. In the QC process 18 HEBCS samples were excluded. The imputation of SUCCESS-A was conducted based on 1000 Genomes Project phase 1 reference haplotypes with program Impute2 with pre-phasing via SHAPEIT2. The imputed data was obtained from dbGaP similarly as the genotyped data. The SUCCESS-A imputed data contained 3,312 samples. The QC of SNPs was performed identically as for the HEBCS and POSH GWS imputed data.

In silico tools and methods

Inspection of haplotypes for the rs992531 locus was performed with program PLINK [8] and R-package haplo.stats. In PLINK we utilized linkage disequilibrium based SNP pruning. The haplotype building was made with R-package haplo.stats. The program LocusZoom was used to plot regional association results [9]. In the Kaplan-Meier Plotter [10] we used the best jetset probe set (an optimal probe set that represents a gene calculated by the jetset program [11]) if there were multiple options for a probe set per gene. For the *RHOBTB2* we used 209441_at probe set and for the *RAB9A* the probe set 221808_at was used. Five-year relapse free survival was used in the gene expression survival analysis in Kaplan-Meier database consistently with the GWAS analysis.

REFERENCES

1. Syrjäkoski K, Vahteristo P, Eerola H, Tamminen A, Kivinummi K, Sarantaus L, Holli K, Blomqvist C, Kallioniemi OP, Kainu T, Nevanlinna H. Population-based study of BRCA1 and BRCA2 mutations in 1035 unselected Finnish breast cancer patients. J Natl Cancer Inst. 2000; 92:1529–31.

2. Kilpivaara O, Bartkova J, Eerola H, Syrjäkoski K, Vahteristo P, Lukas J, Blomqvist C, Holli K, Heikkilä P, Sauter G, Kallioniemi OP, Bartek J, Nevanlinna H. Correlation of CHEK2 protein expression and c.1100delC mutation status with tumor characteristics among unselected breast cancer patients. Int J Cancer. 2005; 113:575–80. https://doi.org/10.1002/ijc.20638.
3. Eerola H, Blomqvist C, Pukkala E, Pyrhönen S, Nevanlinna H. Familial breast cancer in southern Finland: how prevalent are breast cancer families and can we trust the family history reported by patients? Eur J Cancer. 2000; 36:1143–8.
4. Eccles D, Gerty S, Simmonds P, Hammond V, Ennis S, Altman DG. Prospective study of Outcomes in Sporadic versus Hereditary breast cancer (POSH): study protocol. BMC Cancer. 2007; 7:160. https://doi.org/10.1186/1471-2407-7-160.
5. Rafiq S, Tapper W, Collins A, Khan S, Politopoulos I, Gerty S, Blomqvist C, Couch FJ, Nevanlinna H, Liu J, Eccles D. Identification of inherited genetic variations influencing prognosis in early onset breast cancer. Cancer Res. 2013. https://doi.org/0008-5472.CAN-12-3377 [pii]10.1158/0008-5472.CAN-12-3377.
6. Tryka KA, Hao L, Sturcke A, Jin Y, Wang ZY, Ziyabari L, Lee M, Popova N, Sharopova N, Kimura M, Feolo M. NCBI›s Database of Genotypes and Phenotypes: dbGaP. Nucleic Acids Res. 2014; 42:D975–9. https://doi.org/10.1093/nar/gkt1211.
7. Li J, Humphreys K, Heikkinen T, Aittomaki K, Blomqvist C, Pharoah PD, Dunning AM, Ahmed S, Hooning MJ, Martens JW, van den Ouweland AM, Alfredsson L, Palotie A, et al. A combined analysis of genome-wide association studies in breast cancer. Breast Cancer Res Treat. 2011; 126:717–27. https://doi.org/10.1007/s10549-010-1172-9.
8. Purcell S, Neale B, Todd-Brown K, Thomas L, Ferreira MA, Bender D, Maller J, Sklar P, de Bakker PI, Daly MJ, Sham PC. PLINK: a tool set for whole-genome association and population-based linkage analyses. Am J Hum Genet. 2007; 81:559–75. https://doi.org/10.1086/519795.
9. Pruim RJ, Welch RP, Sanna S, Teslovich TM, Chines PS, Gliedt TP, Boehnke M, Abecasis GR, Willer CJ. LocusZoom: regional visualization of genome-wide association scan results. Bioinformatics. 2010; 26:2336–7. https://doi.org/10.1093/bioinformatics/btq419.
10. Gyorffy B, Lanczky A, Eklund AC, Denkert C, Budczies J, Li Q, Szallasi Z. An online survival analysis tool to rapidly assess the effect of 22,277 genes on breast cancer prognosis using microarray data of 1,809 patients. Breast Cancer Res Treat. 2010; 123:725–31. https://doi.org/10.1007/s10549-009-0674-9.
11. Li Q, Birkbak NJ, Gyorffy B, Szallasi Z, Eklund AC. Jetset: selecting the optimal microarray probe set to represent a gene. BMC Bioinformatics. 2011; 12:474. https://doi.org/10.1186/1471-2105-12-474.
